# Dietary vitamin B12 regulates chemosensory receptor gene expression via the MEF2 transcription factor in *Caenorhabditis elegans*

**DOI:** 10.1093/g3journal/jkac107

**Published:** 2022-05-04

**Authors:** Aja McDonagh, Jeannette Crew, Alexander M van der Linden

**Affiliations:** Department of Biology, University of Nevada, Reno, NV 89557, USA; Department of Biology, University of Nevada, Reno, NV 89557, USA; Department of Biology, University of Nevada, Reno, NV 89557, USA

**Keywords:** diet, vitamin B12, *Caenorhabditis elegans*, *srh-234*, chemosensory receptor gene, ADL, sensory neurons, plasticity

## Abstract

Dynamic changes in chemoreceptor gene expression levels in sensory neurons are one strategy that an animal can use to modify their responses to dietary changes. However, the mechanisms underlying diet-dependent modulation of chemosensory gene expression are unclear. Here, we show that the expression of the *srh-234* chemoreceptor gene localized in a single ADL sensory neuron type of *Caenorhabditis elegans* is downregulated when animals are fed a *Comamonas aquatica* bacterial diet, but not on an *Escherichia coli* diet. Remarkably, this diet-modulated effect on *srh-234* expression is dependent on the micronutrient vitamin B12 endogenously produced by *Comamonas aq.* bacteria. Excess propionate and genetic perturbations in the canonical and shunt propionate breakdown pathways are able to override the repressive effects of vitamin B12 on *srh-234* expression. The vitamin B12-mediated regulation of *srh-234* expression levels in ADL requires the MEF-2 MADS domain transcription factor, providing a potential mechanism by which dietary vitamin B12 may transcriptionally tune individual chemoreceptor genes in a single sensory neuron type, which in turn may change animal responses to biologically relevant chemicals in their diet.

## Introduction

Animals receive dietary inputs from their environment and their internal metabolic state, which allows them to modify their chemosensory response properties and behavioral outcomes (Sengupta 2013). One strategy that animals can use to trigger long-term changes in behavioral outcomes is by dynamically changing the expression of individual chemoreceptor genes localized in different chemosensory neuron types. These dynamic transcriptional changes in chemoreceptor gene expression in response to food and internal feeding state is observed in mosquitoes and play pivotal roles in their ability to seek food and reproduce (Fox *et al.* 2001; Hallem *et al.* 2004; Rinker *et al.* 2013; Ryan *et al.* 2014; Taparia *et al.* 2017; Khan *et al.* 2021), but the mechanisms controlling this plasticity in chemoreceptor gene expression are unclear.

The nematode *Caenorhabditis* *elegans* is an excellent model organism to study interactions between an animal and its dietary sources (Yilmaz and Walhout 2014; Zhang *et al.* 2017). *C.* *elegans* is a bacterivore, making it easy to expose these animals to different bacterial strains to study their effects on organismal health and physiology. Bacterially derived factors affect *C. elegans* in various ways; for instance, pathogenic factors are sensed by chemosensory neurons and trigger avoidance behaviors (Pradel *et al.* 2007; Meisel *et al.* 2014), while other bacterially derived factors are innocuous and contribute to physiology and development (Coolon *et al.* 2009; Gracida and Eckmann 2013). Recent work demonstrated that vitamin B12 obtained by *C. elegans* through its bacterial diet is an important nutritional factor in developmental growth and physiology of *C. elegans* (MacNeil *et al.* 2013). The vitamin B12 status of *C. elegans* can be easily assessed with help of the *acdh-1p::GFP* reporter, which is expressed in response to propionate accumulation resulting from B12 deficiency (Watson *et al.* 2013, 2014, 2016). When fed a vitamin B12-deficient *Escherichia* *coli* OP50 diet, *acdh-1* is highly expressed in animals, whereas *acdh-1* is lowly expressed when grown on the vitamin B12-producing *Comamonas aq.* DA1877 diet. The effects of these bacterial diets on *acdh-1* promoter activity have led to important insights into the vitamin B12-dependent and independent propionate breakdown pathways.

*Caenorhabditis* *elegans* is also an ideal organism to study the plasticity in expression levels of individual chemosensory receptor genes in response to external and internal signals (Gruner and van der Linden 2015; Vidal *et al.* 2018). Our prior study showed that the expression levels of the *srh-234* chemoreceptor gene in the ADL sensory neuron type is regulated by starvation. This starvation-mediated modulation of *srh-234* expression levels is dependent on sensory inputs into ADL neurons perceiving food presence, and circuit inputs from RMG interneurons that are electrically connected to ADL perceiving internal state of starvation signals (Gruner *et al.* 2014). Circuit inputs from RMG into ADL regulating *srh-234* required the NPR-1 neuropeptide receptor acting in RMG, as well as insulin signals from other tissues acting on the DAF-2 insulin receptor in ADL (Gruner *et al.* 2014). In addition, starvation-mediated regulation of *srh-234* expression levels in ADL is regulated by both cell- and non-cell-autonomous transcriptional mechanisms involving basic helix-loop-helix (bHLH) factors, including HLH-30 and MXL-3 acting in the intestine, and HLH-2/3 acting together with the MEF-2 factor in ADL neurons (Gruner *et al.* 2016). Together, these findings demonstrated that expression of the *srh-234* chemoreceptor gene in a single ADL sensory neuron type of *C. elegans* is regulated by multiple transcriptional modules, and revealed a neuron-to-intestine connection involving insulin signals in the modulation of chemoreceptor genes as a function of the *C. elegans* feeding state (Gruner and van der Linden 2015).

In this study, we discovered that feeding *C. elegans* vitamin B12-producing *Comamonas aq.* bacteria regulates the expression levels of the *srh-234* chemoreceptor gene in ADL neurons. We show that *srh-234* gene expression is repressed in ADL when animals are fed a high vitamin B12 diet of *Comamonas aq.* DA1877 bacteria relative to a low vitamin B12 diet of *E. coli* OP50 bacteria. This dietary effect of vitamin B12 on *srh-234* in ADL appears to be distinct from the starvation response we reported previously (Gruner *et al.* 2014). Mutant bacteria of *Comamonas aq.* deficient in vitamin B12 production, indicated that *Comamonas*-supplied vitamin B12 regulates *srh-234* expression levels in ADL. Similar to feeding *C. elegans* a *Comamonas aq.* diet, supplementing the *E. coli* diet with exogenous vitamin B12 also represses *srh-234* expression in ADL neurons, in which *E. coli* may function as a vehicle for vitamin B12 uptake by *C. elegans*. The repressing effects of vitamin B12 on *srh-234* expression in ADL neurons can be suppressed by excess propionate supplementation and genetic perturbations in the canonical and shunt propionate breakdown pathways. Lastly, we show that the MEF-2 transcription factor and its binding site in the *srh-234* *cis*-regulatory sequence are necessary for the vitamin B12-mediated repression of *srh-234* expression in ADL. Together, these findings reveal that bacterially derived vitamin B12 turn individual chemoreceptor genes on and off at the level of transcription in sensory neurons that may inform our understanding of how animals fine-tune their chemosensory responses to biologically relevant chemicals in their diet.

## Materials and methods

### *Caenorhabditis elegans* strains and growth conditions

Strains used in this study were: wild-type N2 *C*. *elegans* variety Bristol, RB1774 *pcca-1(ok2282)*, VC1307 *pccb-1(ok1686)*, RB1434 *mmcm-1(ok1637)*, VC1011 *acdh-1(ok1489)*, RB2572 *hphd-1(ok3580)*, RB755 *metr-1(ok521)*, VC1527 *nhr-68(gk708)*, JIN1375 *hlh-30(tm1978)*, and KM134 *mef-2(gv1)*. Transgenic strains used in this study were: VDL3 *oyIs56*[*srh-234p::GFP, unc-122p::RFP*], VDL497 *sanEx497*[*sre-1p::GFP, rol-6(su1006)*], VDL494 *sanEx494*[*sre-1p(+srh-234 MEF2 site)::GFP, rol-6(su1006)*], VDL499 san*Ex499*[*srh-234p(165 bp WT)::GFP, rol-6(su1006)*], VDL299 san*Ex299*[*srh-234p(165 bp -MEF2)::GFP, rol-6(su1006)*], and VL749 *wwIs24*[*acdh-1p::GFP*, *unc-119(+)*]. Animals were cultivated at 20°C on the surface of Nematode Growth Media (NGM) agar. Unless specified otherwise, animals were fed *E. coli* OP50 as the primary food source (Brenner 1974). Genotypes used in this study were confirmed by PCR (e.g. identifying deletions), or by sequencing a PCR product (e.g. identifying single nucleotide changes).

### Bacterial strains and growth conditions

Bacterial strains used in this study were: *E. coli* OP50, *E. coli* HT115 (DE3), *E. coli* HB101, *E. coli* BW25113, *E. coli* Δ*tonB* JW5195, *Comamonas aq.* DA1877, *Comamonas aq.* Δ*cbiA*, and Δ*cbiB* mutants. Bacterial cultures were grown under standard conditions in Luria broth (LB) media until log phase when the optical density (OD) 600 reached approximately 0.6. *Comamonas aq.* mutants were cultured in the presence of 100 μg/ml streptomycin plus 20 μg/ml gentamycin as a selection marker. Presence of these antibiotics did not alter the levels of *srh-234p::GFP* expression. For the killed bacteria experiments, *E. coli* OP50 bacteria were grown to log phase in LB, heat killed at 75°C for 90 min, and added to peptone-free NGM plates.

### Measurement and quantification of promoter::*GFP* reporter expression levels

Animals carrying chemoreceptor::*GFP* reporter genes (i.e. *srh-234, sre-1*) were cultivated at 20°C on NGM plates seeded with *E*. *coli* OP50 as the bacterial food source unless indicated otherwise. Gravid adults were transferred to assay plates and removed after laying eggs. The eggs were then allowed to develop to adults. The increased rate of development when fed *Comamonas aq.* DA1877 was accounted for, and levels of promoter::*GFP* expression of adult animals were then imaged and measured under a microscope equipped with epifluorescence as described previously (Gruner *et al.* 2014, 2016). Briefly, we mounted animals on 2% agarose pads containing 10 mM levamisole, and visualized them on a Leica DM5500 compound microscope equipped with epifluorescence and a Hamamatsu CCD-camera. Microscope and camera settings were kept constant between images of different genotypes and conditions used, unless indicated otherwise. The mean pixel intensity of GFP fluorescence in the entire cell body of ADL was quantified using Volocity software (version 6.3). Prior to measurement, images of ADL cell bodies were cropped for promoter::*GFP* expression level analysis.

### Analysis of *srh-234p::GFP* expression

To analyze *srh-234* expression in mixed bacterial diets, animals carrying the *srh-234p::GFP* reporter were exposed to mixed set ratios, i.e. 1:1, 9:1, and 99:1 ratio of *E. coli* OP50 to *Comamonas aq.* DA1877. To prepare plates, liquid bacterial cultures of OP50 and DA1877 were grown overnight at 37°C in LB and diluted with LB or concentrated to the same OD 600. Bacteria were seeded onto peptone-free NGM agar plates to minimize bacterial growth. Adults expressing the *srh-234p::GFP* reporter were transferred to plates and removed after eggs were laid. Eggs were allowed to develop to adulthood in the presence of the mixed bacterial diets, and *srh-234p::GFP* expression levels were measured and quantified as described above.

To analyze *srh-234* expression in the presence of exogenous vitamin B12 and propionic acid (aka propionate), animals carrying the *srh-234p::GFP* reporter were transferred to NGM plates seeded with *E. coli* OP50 supplemented with or without vitamin B12 (methylcobalamin or Me-Cbl, Sigma, Cat. M9756; adenosylcobalamin or Ado-Cbl, Sigma, Cat. C0884) and propionic acid (Sigma, Cat. P1386). Stocks were made in either ethanol (for Me-Cbl) and water (for Ado-Cbl and propionic acid) to the maximum soluble concentration. Vitamin B12 and propionic acid was diluted to a final 64 nM and 40 mM concentration, respectively, in NGM agar prior to plate pouring. For *E. coli* OP50 supplementation assays with increasing Me-Cbl concentrations, we created a dilution series from a 1-mM Me-Cbl stock. To confirm vitamin B12 action, *acdh-1p::GFP* reporter animals were used as a control in parallel to the *srh-234p::GFP* expression analysis.

For bacterial olfactory assays, we exposed *srh-234p::GFP* reporter animals to a 1-cm^2^ NGM agar square soaked with 1 mM Me-Cbl placed on petri dish lids. For the quadrant petri dish assay, NGM plates were seeded in each quadrant with a 100-μl spot of either *E. coli* OP50 or *Comamonas aq.* DA1877 diets. *srh-234p::GFP* reporter animals were then transferred to a single quadrant of the plate allowing only a single diet for food ingestion, while allowing olfactory cues of the surrounding diets.

### Dye filling of ADL sensory neurons

A stock dye solution prepared in DMSO containing 5 mg/ml red fluorescent lipophilic dye DiI (Sigma, Cat. 42364) was diluted in M9 buffer to a final concentration of 5 μg/ml for optimal signal intensity. Animals carrying the *srh-234p::GFP* reporter (VDL3) were soaked in DiI for 1 h and then rinsed with M9 buffer twice. Stained animals were recovered for 1 h on NGM plates seeded with either *E*. *coli* OP50 or *Comamonas aq.* DA1877 before examination of dye-filled ADL neurons with a Leica DM5500 microscope equipped with epifluorescence.

### Analysis of ADL neuron morphology

To examine the integrity of ADL neurons, animals carrying the integrated *sre-1p:*:*GFP* reporter (VDL497) were cultivated at 20°C on NGM plates seeded with *Comamonas aq.* DA1877. Briefly, we mounted animals on 2% agarose pads containing 10 mM levamisole and visualized them on a Leica Thunder 3D Tissue microscope equipped with a DFC 9000 GT/C sCMOS camera. Z-stack images were obtained at 500 ms exposure time. Small volume computational clearing was applied to the image data set, and a maximum intensity projection was generated.

### Statistical analysis

All results are expressed as means with 95% confidence intervals. Data sets were first analyzed for Gaussian distribution using a normality test (alpha = 0.05, *P* > 0.05) using either the Shapiro–Wilk test or D’Agostino and Pearson normality test to determine whether a parametric or nonparametric statistical test should be performed. Statistical comparisons made for 2 groups include an unpaired *t*-test (parametric) or the Mann–Whitney *t*-test (nonparametric). For more than 2 groups, the ordinary 1-way ANOVA (parametric) or the Kruskal–Wallis test (non-parametric) was used followed by a posthoc multiple comparisons test. Specific statistical tests and *P*-values are reported in the figure legends. All data were graphed and analyzed using Prism 9 software.

## Results

### The *Comamonas aq.* DA1877 diet represses *srh-234* expression in ADL neurons

**Fig. 1.**
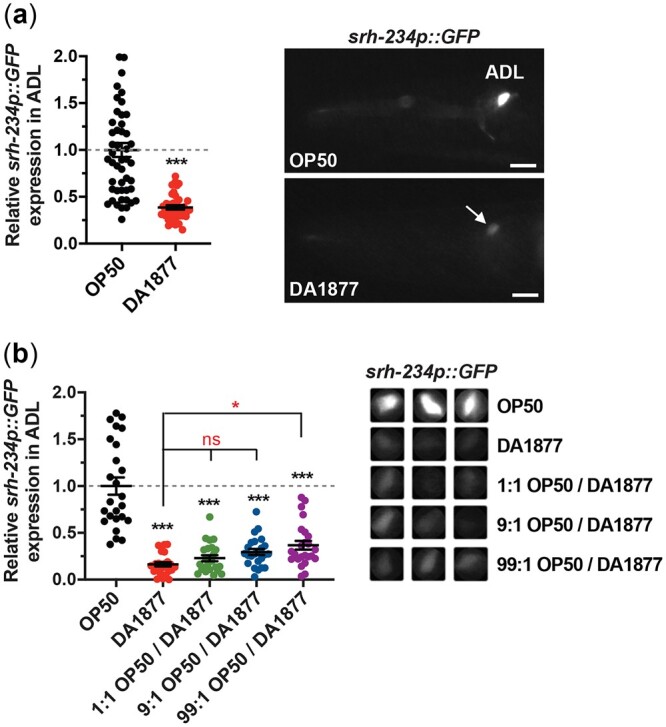
Expression of *srh-234* is repressed by a *Comamonas aq.* DA1877 diet. a) Relative expression levels of *srh-234* in the ADL cell body of adults fed either an *E. coli* OP50 diet or a *Comamonas aq.* DA1877 diet. Adult animals containing stably integrated copies of a *srh-234p::GFP* transgene (*oyIs56*) were examined at the same exposure time on both diets. Images are lateral views of the ADL sensory neuron. Scale is 15 µm. Data are represented as the mean ± SEM (*n* > 38 animals for each diet). ***P < 0.001 with an unpaired 2-tailed *t*-test. b) Relative expression levels of *srh-234* in the ADL cell body of adult animals fed each of the indicated diets. 1:1, 9:1 and 99:1 refers to the dilution of *Comamonas aq.* DA1877 in *E. coli* OP50. Bacteria were seeded on peptone-free plates to prevent bacterial growth (see *Materials and Methods*). Data are represented as the mean ± SEM (*n* > 24 animals for each condition). The Kruskal–Wallis with Dunn multiple-comparisons test was used to determine the statistical significance of differences vs wild-type animals fed *E. coli* OP50, with brackets indicating statistical differences between 2 specific diet conditions. ns, not significant, *P < 0.05. Right panel: Representative cropped images of *srh-234p::GFP* expression in the ADL cell body of adults. Images were acquired at the same exposure time.

To study how bacterial diet regulates chemoreceptor gene expression levels in *C. elegans*, we used the candidate *srh-234* chemoreceptor gene specifically expressed in a single sensory neuron type, ADL. We previously found that GFP expression driven by only 165 bp *cis*-regulatory sequence of *srh-234* (referred here as *srh-234p::GFP*) is rapidly (<1 h) downregulated in starved animals (Gruner *et al.* 2014). While testing the *srh-234p::GFP* reporter in different bacterial diets, we observed that animals fed a *Comamonas aq.* DA1877 diet repress *srh-234* expression in ADL neurons similar to that observed in starved animals (Gruner *et al.* 2014); that is *srh-234p::GFP* expression levels in adult animals is strongly decreased when fed a *Comamonas aq.* DA1877 diet compared to a *E. coli* OP50 diet (Fig. 1a). This *Comamonas*-mediated repression of *srh-234* expression levels is rapid as adult animals raised on *E. coli* OP50 and then transferred to a DA1877 diet decrease *srh-234p::GFP* expression in ADL by 50% about 2 h after the transfer (Supplementary Fig. 1a). Animals fed other *E. coli* diets such as the K12/B-type hybrid HB101 strain, and the K12-type HT115 strain commonly used in *C. elegans* research showed a *srh-234* expression phenotype intermediate to that of *E. coli* OP50 and *Comamonas aq.* DA1877 diets (Supplementary Fig. 1b).

The dietary effect of *Comamonas aq.* DA1877 on *srh-234* expression appears to be distinct from the starvation response, because mixing the *E. coli* OP50 diet with *Comamonas aq.* DA1877 diet 1:1 resulted in animals in which *srh-234* expression levels remained strongly decreased similar to starvation (Fig. 1b). Moreover, smaller concentrations of *Comamonas aq.* DA1877 by diluting it in *E. coli* OP50 (i.e. 9:1 and 99:1 OP50/DA1877) was sufficient to strongly repress *srh-234* expression. Others have reported that *Comamonas aq.* DA1877 bacteria are not a nutrient-poor diet for *C. elegans* (Shtonda and Avery 2006; MacNeil *et al.* 2013), suggesting that *Comamonas aq.* DA1877 may generate a bacterial signal that regulates *srh-234* expression levels. This dietary effect of *Comamonas* on *srh-234* may be specific since expression of another ADL-specific *sre-1* chemoreceptor is not affected (Supplementary Fig. 1c). Since we previously showed that lack of functional ADL cilia can dramatically decrease *srh-234p::GFP* expression levels (Gruner *et al.* 2014), it is possible that the *Comamonas* diet affects the integrity of ADL neurons resulting in the reduced *srh-234* expression. However, we found that wild-type animals fed a *Comamonas aq.* DA1877 diet show normal ADL morphology determined by *sre-1p::GFP* expression (Supplementary Fig. 1, d and e) and wild-type dye filling (Supplementary Fig. 1f) ruling out cilia defects of ADL neurons fed on the *Comamonas aq.* DA1877 diet. Together, these results suggest that in addition to starvation, a dilutable bacterial metabolite produced by *Comamonas aq.* DA1877 bacteria represses *srh-234* expression levels in ADL neurons.

### Vitamin B12 produced by *Comamonas aq.* represses *srh-234* expression

**Fig. 2.**
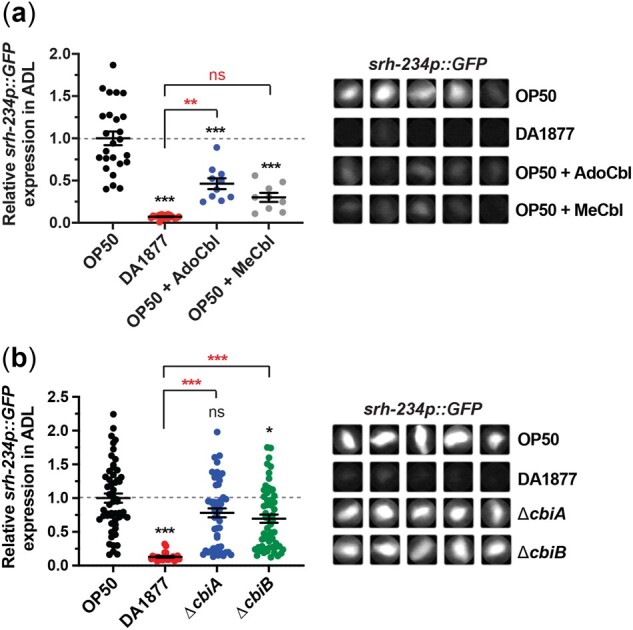
Vitamin B12 produced by *Comamonas aq.* DA1877 is required to repress *srh-234* expression. a) Relative expression of *srh-234* in the ADL cell body of OP50-fed adult animals supplemented with either Ado-Cbl or Me-Cbl compounds at a 64nM final concentration. Data are represented as the mean ± SEM (*n* > 9 animals for each condition). A 1-way ANOVA with Tukey multiple-comparisons test was used to determine the statistical significance of differences vs wild-type animals fed *E. coli* OP50, with brackets indicating statistical differences between 2 specific diet conditions. ns, not significant, **P < 0.01, ***P < 0.001. b) Relative expression of *srh-234* in the ADL cell body of adults fed the *Comamonas aq.* mutant strains Δ*cbiA* and Δ*cbiB* defective in producing vitamin B12 diets compared to *E. coli* OP50 and *Comamonas aq.* DA1877. Data are represented as the mean ± SEM (*n* = 24–56 animals for each condition). The Kruskal–Wallis with Dunn multiple-comparisons test was used to determine the statistical significance of differences vs wild-type animals fed *E. coli* OP50, with brackets indicating statistical differences between 2 specific diet condition. ns, not significant, ***P < 0.001. a, b) Right panels: Representative cropped images of *srh-234p::GFP* expression in the ADL cell body of adults. Images were acquired at the same exposure time for comparison.

The strain *Comamonas aq.* DA1877 produces the dilutable metabolite vitamin B12, while the *E. coli* OP50 strain is not able to synthesize vitamin B12 (Watson *et al.* 2014). To test the hypothesis that vitamin B12 represses *srh-234* expression levels in ADL neurons, we examined *C. elegans* animals fed a *E. coli* OP50 diet supplemented with 2 biologically active and interconvertible forms of vitamin B12, adenosylcobalamin (Ado-Cbl), and methylcobalamin (Me-Cbl). We found that animals fed an *E. coli* OP50 diet supplemented with either 64 nM vitamin B12 (Ado-Cbl or Me-Cbl) repress *srh-234p::GFP* expression in ADL neurons (Fig. 2a). Moreover, supplementing *E. coli* OP50 with increasing concentrations (nM doses) of vitamin B12 (Me-Cbl) resulted in a dose-dependent decrease in *srh-234p::GFP* expression (Supplementary Fig. 2a), which fits with our observation that diluting *Comamonas aq.* DA1877 into the *E. coli* OP50 diet is sufficient to repress *srh-234* expression (Fig. 1b). As a control, we found similar dose-dependent repressive effects of vitamin B12 (Me-Cbl) using the *acdh-1p::GFP* dietary reporter expressed primarily in the intestine (MacNeil *et al.* 2013; Watson *et al.* 2014) when fed either the vitamin B12-producing *Comamonas aq.* DA1877 or the *E. coli* OP50 diet supplemented with vitamin B12 (Me-Cbl) (Supplementary Fig. 2b). These results are also consistent with the observed decrease in *srh-234* expression when animals were fed on *E. coli* HT115 and *E. coli* HB101 diets (Supplementary Fig. 1b), which are better sources of vitamin B12 levels than *E. coli* OP50 (Revtovich *et al.* 2019). Thus, vitamin B12 supplementation to the *E. coli* OP50 diet can repress *srh-234* expression in ADL neurons.

To further test whether *Comamonas*-supplied vitamin B12 regulates *srh-234* expression in ADL, we took advantage of 2 mutant strains of *Comamonas aq.* bacteria (Δ*cbiA* and Δ*cbiB*) that are deficient in vitamin B12 production (Watson *et al.* 2014). We found that feeding animals Δ*cbiA* and Δ*cbiB* bacterial mutants fail to repress *srh-234p::GFP* expression compared to DA1877-fed animals (Fig. 2c). As expected, Δ*cbiA* and Δ*cbiB* bacterial mutants also fail to repress *acdh-1* expression as determined by the *acdh-1p::GFP* reporter (Supplementary Fig. 2d). Together, these findings suggest that vitamin B12 synthesized by *Comamonas aq.* DA1877 bacteria repress *srh-234* expression in ADL.

### *Escherichia coli* may act as a conduit for vitamin B12 uptake by *C*. *elegans*, to in turn regulate *srh-234* expression

**Fig. 3.**
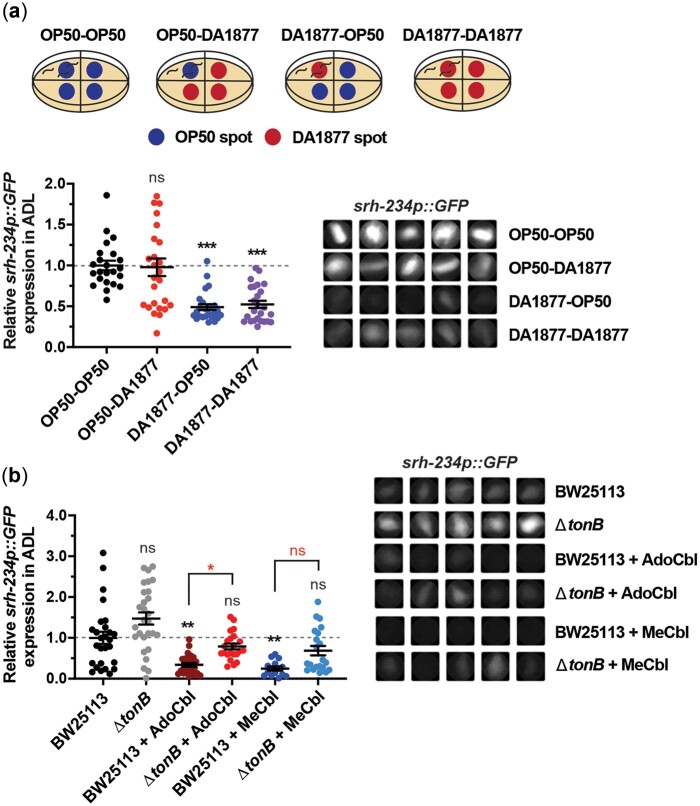
*Escherichia coli* bacteria act as a vehicle for vitamin B12 uptake by *C. elegans*, to in turn regulate *srh-234* expression. a) Top panel: Schematic of a bacterial olfactory assay setup using quadrant assay plates. Adult animals are placed into 1 quadrant allowing only 1 diet to be ingested, while surrounding quadrants are seeded with either the *E. coli* OP50 diet (control) or the *Comamonas aq.* DA1877 diet. Lower panel: Relative expression of *srh-234p::GFP* in the ADL cell body of adults fed on either the *E. coli* OP50 or *Comamonas aq.* DA1877 diet on quadrant plates with surrounding inaccessible diets. Data are represented as the mean ± SEM (*n* > 23 animals for each diet). The Kruskal–Wallis with Dunn multiple-comparisons test was used to determine the statistical significance of differences vs wild-type animals fed *E. coli* (OP50-OP50), with brackets indicating statistical differences between 2 specific conditions. ns, not significant, ***P < 0.001. b) Relative expression of *srh-234* in the ADL cell body of adults fed the *E. coli* Δ*tonB* mutant (strain JW5195) compared to its parental wild-type strain (BW25113) supplemented with or without Ado-Cbl and Me-Cbl (64 nm final concentrations). Data are represented as the mean ± SEM (*n* = 14–28 animals for each condition). The Kruskal–Wallis with Dunn multiple-comparisons test was used to determine the statistical significance of differences vs wild-type animals fed *E. coli* BW25113, with brackets indicating statistical differences between 2 specific bacterial genotypes and conditions. ns, not significant, *P < 0.05, **P < 0.01. Right panel: *srh-234p::GFP* is weakly expressed in adults when fed *E. coli* BW25113 compared to *E. coli* OP50. a, b) Representative cropped images of *srh-234p::GFP* expression in the ADL cell body of adults. Images were acquired at the same exposure time for comparison.

Since *E. coli* OP50 bacteria supplemented with exogenous vitamin B12 can repress *srh-234* expression, we reasoned that *E. coli* may function as a vehicle for the provision and uptake of vitamin B12 by *C. elegans*, to in turn regulate *srh-234* expression in ADL neurons. Alternatively, it remains possible that the availability of vitamin B12 in these supplementation experiments can also be directly perceived by ADL and regulate *srh-234*. To distinguish between these possibilities, we first tested whether vitamin B12 can be perceived as a volatile olfactory cue by ADL neurons to regulate *srh-234*. We exposed animals to live *Comamonas aq.* DA1877 which they cannot eat or touch, while feeding live *E. coli* OP50, and vice versa (Fig. 3a). In addition, we exposed animals expressing *srh-234p::GFP* to plates seeded with live *E. coli* OP50 that were covered with petri-dish lids containing NGM agar squares soaked with 1 mM of vitamin B12 (Me-Cbl) placed above the animals (Supplementary Fig. 3a). In both olfactory assays, we found that *srh-234* expression in ADL was not significantly altered when exposed to volatile cues of *Comamonas aq.* bacteria or the addition of exogenous vitamin B12. Thus, vitamin B12 may not act as a volatile chemical cue to repress *srh-234* expression, but we cannot exclude the possibility that vitamin B12 may still be directly sensed by ADL neurons.

We next tested whether *E. coli* bacteria could act as a vehicle for vitamin B12 uptake by *C. elegans* in vitamin B12 supplementation experiments. The *tonB* transporter in *E. coli* has been shown to be vital for vitamin B12 uptake from its extracellular environment (Bassford *et al.* 1976; Kadner 1990). Recent work showed that *C. elegans* animals fed an *E. coli* BW25113 diet in which the *tonB* transporter gene is deleted (Δ*tonB*) can block, at least in part, the repression of the *acdh-1p::GFP* reporter when the growth media is supplemented with vitamin B12 (Revtovich *et al.* 2019). Based on these observations, we hypothesized that the effects of vitamin B12 on repressing *srh-234* expression would also be dependent on the *E. coli* TonB pathway. We found that supplementing the *E. coli* BW25113 diet with vitamin B12 (Ado-Cbl or Me-Cbl) repressed *srh-234p::GFP* expression similar to that observed for *E. coli* OP50-fed animals supplemented with vitamin B12 (Fig. 3b). When *tonB* was deleted (Δ*tonB*), we found a small, but statistically significant (*P < *0.05) block in the repression of *srh-234* by Ado-Cbl but less so with Me-Cbl (Fig. 3b). Thus, *E. coli* bacteria via the TonB transporter may be an important determinant for the vitamin B12 uptake by *C. elegans* to in turn regulate *srh-234* expression. However, because Δ*tonB* did not completely block the effects of vitamin B12 on repressing *srh-234*, it is likely that there are alternative TonB independent routes to obtain vitamin B12 from the media.

To further validate that *E. coli* bacteria acts as a vehicle for vitamin B12 uptake by *C. elegans*, we asked whether heat-killed *E. coli* bacteria supplemented with vitamin B12 could repress *srh-234* expression. Growing worms on heat-killed *E. coli* bacteria is challenging, because heat destroys some of the nutrients needed for worms to develop and renders the bacteria less edible, resulting in larvae that arrest their development (Qi *et al.* 2017). We therefore exposed animals to live *E. coli* OP50 until adulthood, and then transferred young adults to plates seeded with *E. coli* OP50 killed by high heat (>75 °C) with and without vitamin B12 (Me-Cbl). Adults fed heat-killed *E. coli* OP50 without vitamin B12 (HK-OP50) grew slowly as expected and resulted in animals with significantly reduced *srh-234p::GFP* expression 48 h after their transfer (Supplementary Fig. 3b), mimicking the starvation-induced reduction in *srh-234* expression we observed previously (Gruner *et al.* 2014). However, when we fed adults heat-killed *E. coli* OP50 bacteria with vitamin B12 (HK-OP50+B12), the vitamin B12 supplementation had a beneficial effect as worms grew faster consistent with previous observations (Qi *et al.* 2017), but *srh-234p::GFP* expression was less repressed by vitamin B12 compared to live *E. coli* OP50 with vitamin B12 (OP50+B12) (Supplementary Fig. 3b). We found similar results for the *acdh-1::GFP* dietary reporter (Supplementary Fig. 3b).

Thus, consistent with our TonB findings, *E. coli* bacteria in our vitamin B12 supplementation experiments may merely act as a vehicle for vitamin B12 uptake by *C. elegans*, which in turn can repress the expression of *srh-234* in ADL neurons.

### Propionate overrides the repressing effects of vitamin B12 on *srh-234* expression

**Fig. 4.**
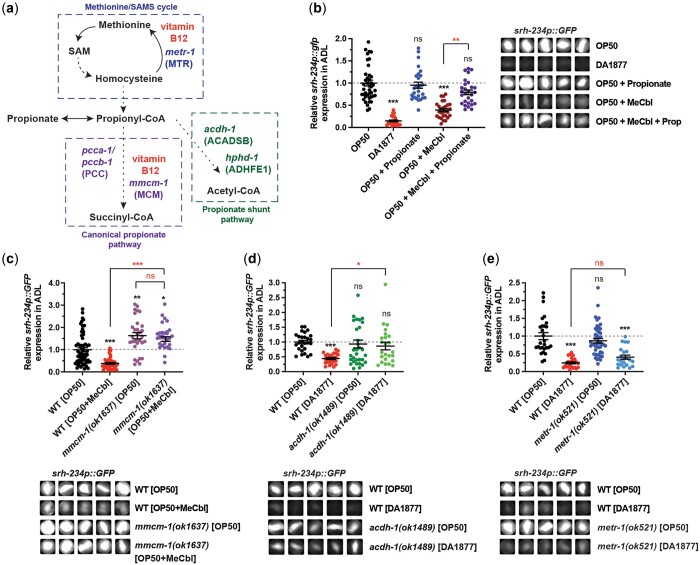
The repressive effects of vitamin B12 on *srh-234* expression are reversed by propionate accumulation. a) Schematic of the *C. elegans* vitamin B12-dependent methionine/SAMs cycle, the vitamin B12-dependent canonical propionate breakdown pathway, and the propionate shunt pathway independent of vitamin B12. Genes shown are: *metr-1*/MTR (methionine synthase), *pcca-1*/PCCA and *pccb-1*/PCCB (2 members of the propionyl-CoA carboxylase complex), *mmcm-1*/MCM (methylmalonyl-CoA mutase), *acdh-1*/ACADSB (acyl-CoA dehydrogenase), and *hphd-1*/ADHFE1 (3-hydroxypropionate-oxoacid transhydrogenase). b) Relative expression of *srh-234* in the ADL cell body of adults fed an *E. coli* OP50 diet supplemented with excess propionate (40 mM final concentration) and/or Me-Cbl (64 nM final concentration). Data are represented as the mean ± SEM (*n* = 24–42 animals for each condition). A 1-way ANOVA with Tukey multiple-comparisons test was used to determine the statistical significance of differences vs wild-type animals fed *E. coli* OP50, with brackets indicating statistical differences between 2 specific diet conditions. ns, not significant, **P < 0.01, ***P < 0.001. c–e) Relative expression of *srh-234* in the ADL cell body of adult animals with mutations in the canonical propionate breakdown pathway (c), the propionate shunt breakdown pathway (d), and the methionine/SAM cycle (e) fed *E. coli* OP50 with or without exogenous vitamin B12 (Me-Cbl, 64 nM), or *Comamonas aq.* DA1877. Data are represented as the mean ± SEM. *n* > 18 animals for each diet and genotype. c–e) The Kruskal–Wallis with Dunn multiple-comparisons test was used to determine the statistical significance of differences vs wild-type animals fed *E. coli* OP50, with brackets indicating statistical differences between 2 specific genotypes and conditions. ns, not significant, *P < 0.05, **P < 0.01, ***P < 0.001. b–e) Representative cropped images of *srh-234p::GFP* expression in the ADL cell body of adults. Images were acquired at the same exposure time for comparison.

In the presence of vitamin B12 when fed a *Comamonas aq.* DA1877 diet, *C. elegans* preferably uses the canonical vitamin B12-dependent propionate breakdown pathway (Fig. 4a). However, in low dietary vitamin B12 conditions when fed an *E. coli* OP50 diet, *C. elegans* mainly utilizes and transcriptionally activates its alternative vitamin B12 independent propionate shunt pathway to breakdown toxic propionate buildup (Watson *et al.* 2013, 2014, 2016) (Fig. 4a). The balance between vitamin B12 and propionyl-CoA levels involved in this propionate breakdown controls promoter activity of the *acdh-1* gene; that is excess propionate is able to override the repressing effects of vitamin B12 on *acdh-1* expression (Watson *et al.* 2016). Similarly, we found that animals fed an *E. coli* OP50 diet supplemented with both vitamin B12 (Me-Cbl, 64 nM) and excess propionate (40 mM) can override the repressing effects of vitamin B12 on *srh-234p::GFP* expression (Fig. 4b).

Genetic perturbation of the canonical and propionate shunt breakdown pathway can also lead to propionate accumulation, such that loss of both *pcca-1* and *acdh-1* function results in synthetic lethality likely due to toxic effects of propionate buildup (Watson *et al.* 2013, 2014, 2016). We therefore tested whether propionate buildup due to genetic perturbations in these propionate breakdown pathways also lead to changes in *srh-234* promoter activity. We found that expression of *srh-234p::GFP* in mutants of *mmcm-1* encoding the methylmalonyl-CoA mutase that requires vitamin B12 as a cofactor is not significantly different in animals fed *E. coli* OP50 with or without exogenous vitamin B12 (Me-Cbl) (Fig. 4c). Thus, consistent with excess propionate overriding the effects of vitamin B12 on *srh-234* expression, *mmcm-1* mutations also block the repressive effects of vitamin B12. Similarly, mutations in *pcca-1* and *pccb-1* genes (Supplementary Fig. 4, a and b) or in propionate shunt pathway genes, *acdh-1* and *hphd-1* (Fig. 4d and Supplementary Fig. 4c) can also, at least in part, block the repressing effects of vitamin B12 on *srh-234* when fed on *Comamonas aq.* DA1877. Interestingly, *srh-234p::GFP* expression is slightly increased in mutants of *mmcm-1*, *pccb-1* and *pcca-1* compared to wild-type animals when fed on *E. coli* OP50, which could be explained by a further accumulation of propionate in these conditions. Mutations in the methionine/SAM cycle gene, *metr-1*, did not show significant differences in *srh-234p::GFP* expression compared to wild-type animals when fed on *Comamonas aq.* DA1877 (Fig. 4e). All mutants tested showed a normal dye filling of ADL neurons (Supplementary Fig. 4d). These findings suggest that mainly genetic perturbations in the canonical and propionate shunt breakdown pathways can alter *srh-234* expression levels in ADL.

The nuclear hormone receptor, *nhr-68*, is involved in activating shunt gene expression in the *C. elegans* intestine in response to excessive propionate levels, and mutations in *nhr-68* are proposed to mitigate propionate toxicity (Bulcha *et al*. 2019). We found that animals with mutations in *nhr-68* show reduced *srh-234p::GFP* expression when fed *E. coli* OP50 supplemented with or without exogenous vitamin B12 (Me-Cbl) compared to wild-type animals (Supplementary Fig. 4e). Feeding *nhr-68* mutants *E. coli* OP50 supplemented with both vitamin B12 (Me-Cbl) and excess propionate (40 mM) resulted in significantly higher *srh-234p::GFP* expression levels (*P < *0.0001) than compared to wild-type (Supplementary Fig. 4e). That is, adding excess propionate to *nhr-68* mutants could modestly override the repressing effects of vitamin B12 on *srh-234* expression, but not to the same extent as in wild-type animals. Thus, *nhr-68* is an important determinant in mitigating the response to excessive propionate levels on *srh-234* expression.

Together, these findings suggest that excess propionate can override the repressing effects of vitamin B12 on *srh-234* expression in ADL neurons.

### MEF-2 is required for the vitamin B12-mediated repression of *srh-234* expression

**Fig. 5.**
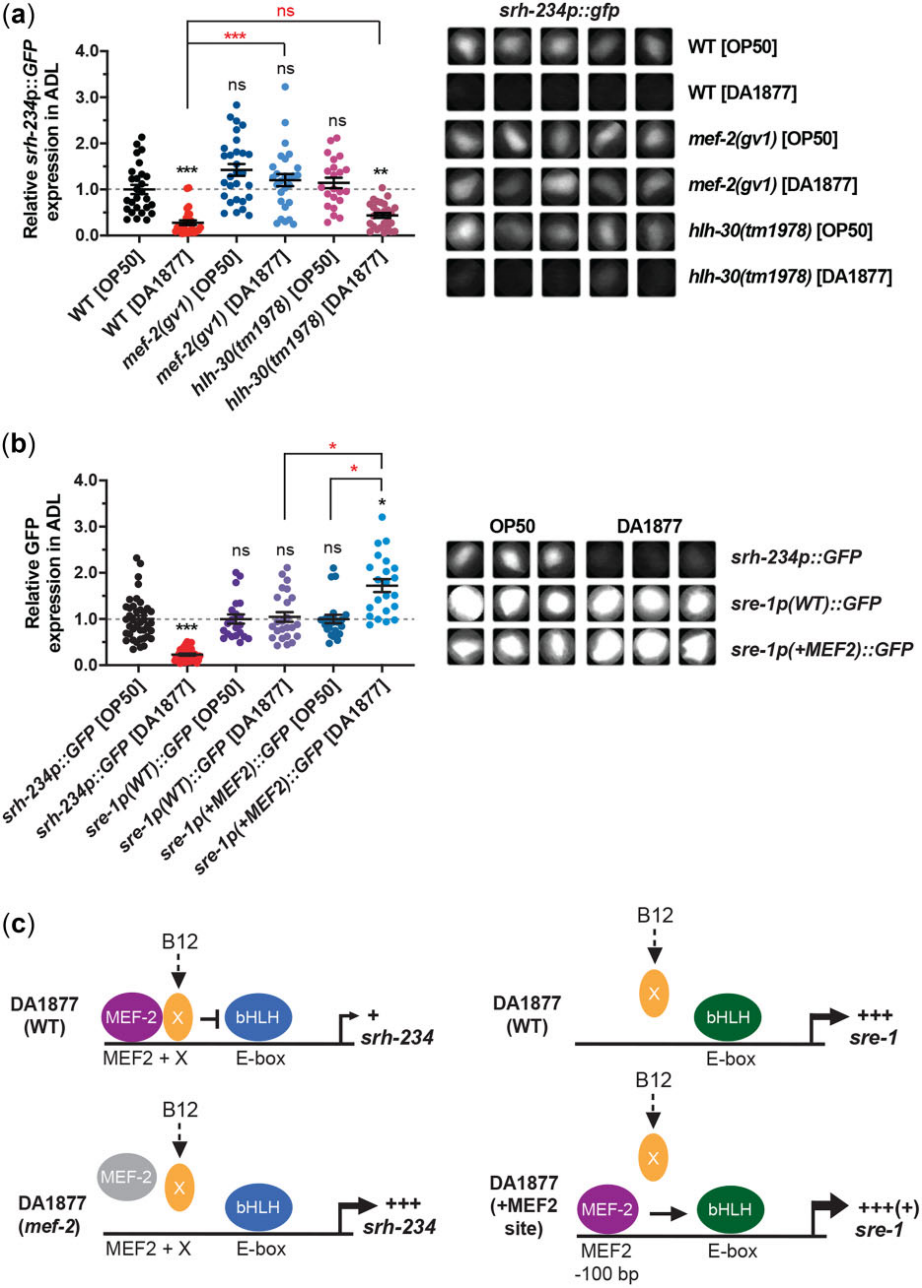
*mef-2* is required for vitamin B12-dependent regulation of *srh-234.* a) Relative expression of *srh-234* in the ADL cell body of *mef-2* and *hlh-30* mutants when adults are fed a *Comamonas aq.* DA1877 diet compared to an *E. coli* OP50 diet. Data are represented as the mean ± SEM (*n* > 22 animals). The Kruskal–Wallis with Dunn multiple-comparisons test was used to determine the statistical significance of differences vs wild-type animals fed *E. coli* OP50, with brackets indicating statistical differences between 2 specific genotypes and conditions. ns, not significant, **P < 0.01, ***P < 0.001. Right panel: Representative cropped images of *srh-234p::GFP* expression in the ADL cell body. Images were acquired at the same but at a lower exposure time. b) Relative expression of wild-type *sre-1p::GFP* (*sre-1p(WT)::GFP*) or the *sre-1* promoter with the inserted MEF2 binding site sequence from the *srh-234* promoter (*sre-1p(+MEF2)::GFP*) in the ADL cell body of adults fed a *Comamonas aq.* DA1877 diet compared to an *E. coli* OP50 diet. Data are represented as the mean ± SEM (*n* = 21–40 animals). For *sre-1* expression, data were normalized to the *sre-1p::GFP* reporter for animals fed *E. coli* OP50. For *srh-234* expression, data were normalized to *srh-234p::GFP* for animals fed *E. coli* OP50. The Kruskal–Wallis with Dunn multiple-comparisons test was used to determine the statistical significance of differences vs wild-type animals fed *E. coli* OP50, with brackets indicating statistical differences between 2 specific genotypes and conditions. ns, not significant, *P < 0.05, ***P < 0.001. Right panel: Representative cropped images of GFP expression under control of the *srh-234* or *sre-1* promoter with or without the *srh-234* MEF-2 binding site in the ADL cell body. Images were acquired at the same exposure time. c) Model based on findings shown in panel (b) explaining the observed expression changes for *srh-234* in *mef-2* mutants, and *sre-1* with a *srh-234* MEF2-binding site artificially inserted in its promoter upstream and close to the identified E-box that drives *sre-1* expression in the ADL neuron. +, +++, and +++(+) indicates low, high, and highly increased expression levels, respectively.

To further interrogate the mechanisms underlying the vitamin B12-dependent regulation of *srh-234* gene expression in ADL neurons, we examined candidate components and pathways. We previously reported that the MEF-2 transcription factor acts together with bHLH factors to regulate the starvation-dependent regulation of *srh-234* expression (Gruner *et al.* 2016). In this mechanism, MEF-2 acts cell-autonomously with bHLH factors HLH-2/HLH-3 in ADL neurons, while HLH-30 and MLX-3 bHLH factors function in the intestine to non-cell-autonomously regulate *srh-234* expression in ADL in response to starvation signals. We found that a mutation in *mef-2* but not in *hlh-30* can fully suppress the vitamin B12-dependent reduction in *srh-234* expression when animals were fed a *Comamonas aq.* DA1877 diet, suggesting that the MEF-2 factor is required for the vitamin B12-dependent regulation of *srh-234* (Fig. 5a and Supplementary Fig. 5a). We next took advantage of *srh-234p(-MEF-2)::GFP* reporter animals in which the core of the MEF2 binding in the *srh-234* promoter is mutated (Gruner *et al.* 2016), and found that vitamin B12-supplemented *E. coli* OP50 bacteria fail to repress *srh-234p(-MEF-2)::GFP* expression compared to *srh-234p(WT)::GFP* expressing animals (Supplementary Fig. 5b). These results suggest that the MEF-2 factor and its binding site in the *srh-234* promoter are necessary for the vitamin B12-dependent repression of *srh-234* expression.

We next examined whether the MEF-2 binding site in the *srh-234* promoter was sufficient for the vitamin B12-dependent regulation of *srh-234*. To test this, we used a transgenic reporter strain of the *sre-1* promoter fused to *gfp* with or without the MEF-2 binding site identified in the *srh-234* promoter (AGTTATATTTAA) (Supplementary Fig. 5c) (Gruner *et al.* 2016). The *sre-1* promoter is specifically and highly expressed in ADL neurons, but levels of *sre-1* expression are not significantly changed in animals fed the vitamin B12-producing *Comamonas aq.* DA1877 bacteria (Supplementary Fig. 1c). Surprisingly, we found that animals carrying a transgene of the *sre-1* promoter with the inserted *srh-234* MEF-2 site (*sre-1p(+MEF2)::GFP*) showed similar *sre-1* expression levels in ADL neurons when fed *Comamonas aq.* DA1877 compared to wild-type *sre-1p::GFP* animals (*sre-1p(WT)::GFP*) on the same diet (Fig. 5b). These results suggest that in contrast to the starvation-dependent regulation of *srh-234* (Gruner *et al.* 2014), insertion of the MEF2 binding site alone is not sufficient for the vitamin B12-dependent regulation of *srh-234* expression levels in ADL neurons, suggesting the requirement of another yet unknown factor that may act together with the MEF-2 factor (Fig. 5c).

Taken together, these findings show that the repressing effects of vitamin B12 on the expression of *srh-234* in ADL neurons are dependent on the MEF-2 transcription factor.

## Discussion

**Fig. 6.**
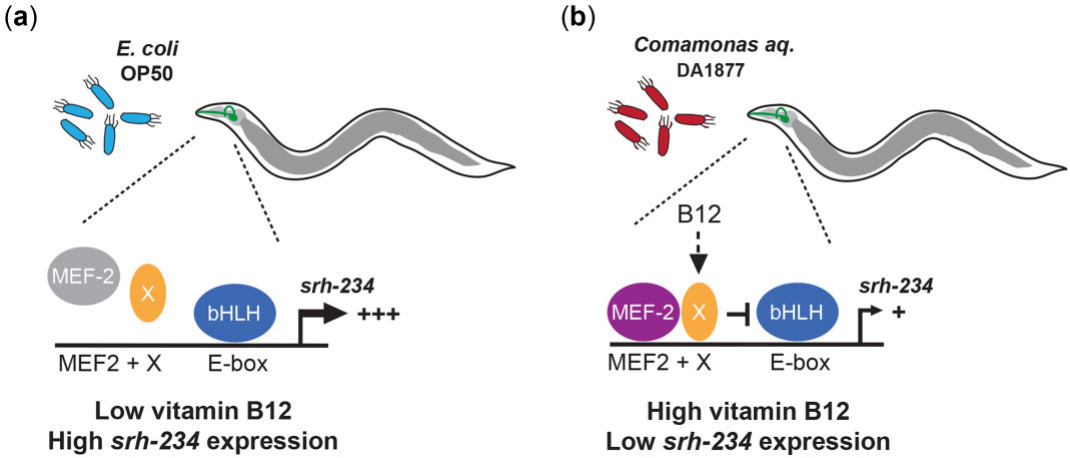
Model for the regulation of *srh-234* chemoreceptor expression levels in the ADL sensory neuron under different dietary conditions. Expression levels of *srh-234* in *C. elegans* animals is high (+++) when fed a low vitamin B12 diet of *E. coli* OP50 bacteria (a) but low (+) when fed a high vitamin B12 diet of *Comamonas aq.* DA1877 bacteria (b). An unknown factor (X) may act together with the MEF-2 transcription factor to repress *srh-234* expression levels under conditions of high vitamin B12 via a bHLH/E-box module important to promote expression of *srh-234* in ADL.

In this study, we show that the expression levels of the *srh-234* chemoreceptor gene in the ADL sensory neuron type is regulated by dietary vitamin B12. In a low vitamin B12 *E. coli* diet, *srh-234* is highly expressed in ADL but not when *C. elegans* is fed a high vitamin B12-producing *Comamonas aq.* DA1877 diet (Fig. 6). Excess propionate and genetic perturbations in the propionate breakdown pathways are able to override the repressing effects of vitamin B12 on *srh-234* expression. In addition, the vitamin B12-mediated regulation of *srh-234* is dependent on the MEF-2 transcription factor. The mechanisms by which dietary vitamin B12 transcriptionally tunes *srh-234* could provide *C. elegans* the means to modify long-term changes in ADL-mediated responses.

This study complements our previous work (Gruner *et al.* 2014, 2016), which explored the dynamics in *srh-234* expression upon starvation, which was dependent on MEF-2 function and its respective MEF2 binding site present in the *cis*-regulatory sequence of *srh-234*. Similarly, we show that loss of MEF-2 function can inhibit the repression of *srh-234* expression in ADL in response to vitamin B12 produced by *Comamonas aq.* DA1877 diets, suggesting that MEF-2 has dual roles in regulating *srh-234* expression in response to both starvation and dietary vitamin B12. However, unlike starvation (Gruner *et al.* 2016), artificial introduction of the *srh-234* MEF2 binding site into the *cis*-regulatory sequence of the *sre-1* gene close to its E-box site that drives expression in ADL neurons (McCarroll *et al.* 2005) did not confer vitamin B12-induced repression via MEF-2. Instead, insertion of the *srh-234* MEF-2 site in the *sre-1* promoter showed higher *sre-1* expression levels when fed the high vitamin B12 *Comamonas aq.* DA1877 diet compared to the low vitamin B12 *E. coli* diet. One possible explanation for the increased *sre-1* expression levels in ADL is that MEF-2 acts to promote (or enhance) *sre-1* transcription via its respective bHLH/E-box module after introducing the MEF2 site near the *sre-1* E-box site. In mammals, it is known that MEF2 factors can act either to activate or repress transcription depending on its interacting cofactors (Molkentin *et al.* 1995; Black *et al.* 1996). Thus, MEF-2 may act to repress or promote expression based on the different bHLH factors that drive *srh-234* and *sre-1* transcription in ADL via their respective E-box sites contained in their promoters (McCarroll *et al.* 2005; Gruner *et al.* 2016).

Based on these findings, we propose a model (Fig. 6) in which animals fed a high vitamin B12 *Comamonas aq.* DA1877 diet regulates *srh-234* expression via a transcriptional module consisting of a MEF-2 factor and its respective MEF2 binding site, together with a yet unknown factor (X) stimulated by dietary vitamin B12. This in turn may repress bHLH factors through an E-box site that promotes *srh-234* in ADL neurons via a complex mechanism involving a combination of different bHLH heterodimer pairs (Gruner *et al.* 2016). When animals are fed a low vitamin B12 diet of *E. coli* OP50, MEF-2 activity no longer represses *srh-234* expression in ADL. Thus, MEF-2 activity is necessary to repress *srh-234* in response to vitamin B12, but the exact nature of factor X that may act with MEF-2 remains to be determined.

In *C. elegans*, vitamin B12 is exclusively obtained from its diet, and our findings support that vitamin B12 produced by *Comamonas aq.* can repress *srh-234* expression in ADL neurons. We further showed that *E. coli* bacteria via the TonB transporter may function as a vehicle for vitamin B12 uptake by *C. elegans*, and its uptake in turn can regulate *srh-234*. However, *tonB* mutations did not completely block the repression of *srh-234* expression when vitamin B12 was added to the growth media. These findings suggest that *E. coli* may have alternative *tonB* independent routes to obtain vitamin B12 from the media consistent with previous reports using the *acdh-1p::GFP* reporter (Revtovich *et al.* 2019). Alternatively, the increased availability of exogenous vitamin B12 added to the media may be directly perceived by ADL sensory neurons, which could play additional roles in the repression of *srh-234* expression in ADL. Using olfactory assays, we showed that vitamin B12 does not appear to act as a volatile chemical cue to regulate *srh-234* in ADL neurons, but we cannot exclude the possibility that vitamin B12 is also directly sensed by ADL in the vitamin B12 supplementation experiments. Our previous work showed that elimination of all sensory inputs into ADL neurons strongly decrease *srh-234* expression in *E. coli* OP50-fed conditions similar to the effects of vitamin B12 (Gruner *et al.* 2014; Gruner and van der Linden 2015), and thus it would be challenging to investigate whether physical or genetic manipulations of ADL sensory dendrites and/or cilia important for sensing the environment can alter the effects of vitamin B12 on *srh-234* expression. Future studies investigating intracellular calcium dynamics in ADL in the presence of vitamin B12 using a genetically encoded Ca^2+^ sensor have the potential to reveal whether ADL neurons can also directly sense vitamin B12.

The balance between vitamin B12 and propionate levels in *C. elegans* is important for tuning *srh-234* promoter activity in ADL neurons. Mutations in the canonical (*pcca-1*, *pccb-1*, and *mmcm-1*) and the shunt propionate (*acdh-1*, *hphd-1*) breakdown pathways, the propionate persistence detector (*nhr-68*), as well as propionate supplementation, were all able to override the repressing effects of vitamin B12 on *srh-234* expression. In mammalian models of propionic acidemia, animals lacking the propionyl CoA-carboxylase (PCCA) were found to have elevated propionate levels shortly after birth (Miyazaki *et al.* 2001). Similarly, *pcca-1-*mutant animals in *C. elegans* may have naturally elevated propionate levels that cannot be restored to normal levels by vitamin B12 sufficient diets alone (Watson *et al.* 2016). Consistent with a persistent accumulation of propionate in *C. elegans* in regulating *srh-234* promoter activity, we show that mutants of *mmcm-1*, *pcca-1*, *pccb-1* significantly enhance the levels of *srh-234* expression on an *E. coli* OP50 diet that is unable to efficiently breakdown propionate by the canonical pathway. Conversely, *srh-234* expression levels are strongly reduced in ADL when exposed to low propionate levels; for instance, in animals that are food deprived (starved) or exposed to high vitamin B12 conditions. Studies in rats demonstrated that after 2 days of starvation, propionate levels are rapidly decreased but again restored after refeeding (Illman *et al.* 1986).

What are the functional consequences of the regulation of chemoreceptor gene expression by dietary-supplied vitamin B12? The nociceptive ADL neuron where *srh-234* is specifically expressed mediates avoidance responses to a wide variety of environmental signals such as odors (Troemel *et al.* 1995, 1997; Chao *et al.* 2004), pheromones (Jang *et al.* 2012), and heavy metals (Sambongi *et al.* 1999; Wen *et al.* 2020). Since chemoreceptor genes expressed in a specific sensory neuron type are generally linked to a common chemical response determined by the identity of the neuron in *C. elegans*, with a few exceptions in which neurons switch their preference toward odors (Tsunozaki *et al.* 2008), it is probable that the *srh-234* chemoreceptor may detect aversive stimuli perceived by ADL. Interestingly, vitamin B12 in mammals has anti-nociceptive properties (Erfanparast *et al.* 2014), and the activity of certain olfactory receptors in tissues other than neurons can respond to propionate (Pluznick *et al.* 2013), which is a metabolic byproduct produced by gut bacteria in mammals (Morrison and Preston 2016). It is therefore tempting to speculate that dietary-supplied vitamin B12 can alter ADL-mediated nociceptive responses to specific cues by changing the expression levels of chemoreceptor genes. However, nothing is known about whether vitamin B12 or propionate levels affects ADL-mediated responses in *C. elegans*. Other than growth, development, and lifespan (Bito *et al.* 2013; MacNeil *et al.* 2013), only recently vitamin B12 in the diet has been reported to be an important micronutrient in the regulation of predatory behaviors between nematodes (Akduman *et al.* 2020).

In summary, the *srh-234* chemoreceptor gene is one of a large repertoire of over 1,300 chemoreceptor genes (Robertson 2000), many of which are localized in a relatively small subset of chemosensory neurons (Vidal *et al.* 2018), such that each neuron expresses multiple chemoreceptor genes. We show here dynamic changes in the expression levels of the *srh-234* chemoreceptor gene localized in the ADL sensory neuron type in response to changing dietary vitamin B12. Other studies have illustrated that dynamic expression changes in individual chemoreceptor genes can have profound effects on behavioral outcomes. For instance, changes in the expression levels of the *odr-10* olfactory receptor required to sense diacetyl (Sengupta *et al.* 1996) in the male *C. elegans* contributes to its plasticity in food detection and feeding/exploration decisions in order to locate mates (Ryan *et al.* 2014). Further research will determine what the functional consequences are of the plasticity in *srh-234* chemoreceptor gene expression in ADL neurons in response to dietary vitamin B12.

## Data availability

All data are available as part of this manuscript. Strains and plasmids are available upon request. Supplementary data and information are available online.

Supplemental material is available at *G3* online.

## Supplementary Material

jkac107_Supplementary_Data
