# Correction to: Dietary vitamin B12 regulates chemosensory receptor gene expression via the MEF2 transcription factor in *Caenorhabditis elegans*

**DOI:** 10.1093/g3journal/jkad111

**Published:** 2023-05-31

This is a correction to: Aja McDonagh and others, Dietary vitamin B12 regulates chemosensory receptor gene expression via the MEF2 transcription factor in *Caenorhabditis elegans, G3 Genes|Genomes|Genetics*, Volume 12, Issue 6, June 2022, jkac107, https://doi.org/10.1093/g3journal/jkac107

In the originally published version of this manuscript, the ‘Received date’ was inadvertently given as October 04, 2022, instead of October 04, 2021.

This error has been corrected.

