# Supplementary material for: Dietary vitamin B12 regulates chemosensory receptor gene expression via the MEF2 transcription factor in Caenorhabditis elegans
Source: G3 (Bethesda). 2022 May 4;12(6):jkac107. doi: 10.1093/g3journal/jkac107 (PMC9157118; doi:10.1093/g3journal/jkac107)
Supplement: jkac107_Supplementary_Data [file jkac107_supplementary_data.docx]

**SUPPLEMENTAL DATA**

**Dietary vitamin B12 regulates chemosensory receptor gene expression via the MEF2 transcription factor in *Caenorhabditis elegans***

Aja McDonagh^1^, Jeannette Crew^1^, and Alexander M. van der Linden^#1^

^1^, Department of Biology, University of Nevada, Reno, Nevada, 89557

^#^, corresponding author

Correspondence to Alexander van der Linden: [avanderlinden@unr.edu](mailto:avanderlinden@unr.edu)

**

**

**Supplemental Figure 1: *srh-234* expression in ADL neurons is rapidly downregulated in the presence of *Comamonas aq.* DA1877.**

**(A)** Time-course of the relative *srh-234p::GFP* expression levels in the ADL cell body of adult animals when transferred from an *E. coli* OP50 diet to a *Comamonas* DA1877 diet. Expression of *srh-234p::GFP* in adults was measured every 15 min at the same exposure time. The graph shows the mean ± SEM (n=5 animals for each timepoint). Right panel: Representative cropped images of *srh-234p::GFP* expression in the ADL cell body in adult animals fed DA1877 at the indicated exposure times. **(B)** Relative expression levels of *srh-234p::GFP* in the ADL cell body of adults fed *E. coli* HB101 and HT115 diets compared to *E. coli* OP50 and *Comamonas aq.* DA1877 diets. Data are represented as the mean ± SEM (n>25 animals for each diet). The Kruskal-Wallis with Dunn multiple-comparisons test was used to determine the statistical significance of differences vs. wild-type animals fed *E. coli* OP50, ** *p*<0.01, *** *p*<0.001. Lower panel: Representative cropped images of *srh-234p::GFP* expression in the ADL cell body of adults fed different diets. **(C)** Relative expression of *sre-1p::GFP* in the ADL cell body of adult animals fed OP50 and DA1877 diets. Animals contain stably integrated copies of a *sre-1p::GFP* transgene (*otIs24*). Data are represented as the mean ± SEM (n>25 animals). ns, not significant by an unpaired 2-tailed *t*-test. Lower panel: Representative cropped images of *sre-1p::GFP* expression in the ADL cell body of adults fed OP50 and DA1877 diets. **(D)** Representative images of *sre-1p::GFP* expression of OP50- and DA1877-fed animals are a ventral view showing the left and right ADL neuron (anterior is left), and images were acquired at the same exposure time. Scale is 15 µm. **(E)** Representative z-stack image of *sre-1p::GFP* expression of DA1877-fed animals with ADL morphological structures labeled. An enlarged inset image of ADL cilia is shown. The image was captured using a Leica 3D Tissue Thunder microscope with small volume computational clearing. Scale is 15 µm. **(F)** Percentage of adult animals with wild-type dye-filling of ADL neurons on OP50 and DA1877 diets. **(A-C)** Images were acquired at the same exposure time for comparison.

**

**

**Supplemental Figure 2: Dose dependent regulation of *srh-234* expression by vitamin B12.**

**(A-B)** Dose-dependent decrease in *srh-234p::GFP* expression in the ADL cell body **(A)** and *acdh-1p::GFP* expression in the intestine **(B)** of *E. coli* OP50-fed animals supplemented with increasing concentrations of Me-Cbl. The graph shows the mean ± SEM (n>12 animals for each time-point). A 2-way ANOVA with Tukey multiple-comparisons test was used to determine the statistical significance of differences vs. wild-type animals fed *E. coli* OP50, *** *p*<0.001. Right Panel: Representative cropped images of *srh-234p::GFP* expression in the ADL cell body acquired at the same exposure time. The dietary sensor *acdh-1p::GFP* was used as a positive control for the action of different Me-Cbl concentrations **(B)** and when animals were fed Δ*cbiA* and Δ*cbiA* mutants of *Comamonas aq.* **(C).** Representative images were acquired at the same exposure time for comparison unless indicated otherwise, and insets in panel B are images taken at a higher exposure time.

**

**

**Supplemental Figure 3: Vitamin B12 does not act as a volatile cue to alter *srh-234* expression levels in ADL sensory neurons**

**(A)** Relative expression of *srh-234p::GFP* in the ADL cell body of adult animals fed *E. coli* OP50 in the presence 1 mM Me-Cbl placed on the cover of the petri dish lid. Data are represented as the mean ± SEM (n>25 animals). ns, not significant by an unpaired 2-tailed *t*-test. Right panel: Representative cropped images of *srh-234p::GFP* expression in the ADL cell body of adults. Images were acquired at the same exposure time. **(B)** Relative expression of *srh-234* in the ADL cell body of adults that are fed 48 hours with either live or heat-killed *E. coli* OP50 supplemented with vitamin B12 (Me-Cbl, 64 nM). Data are represented as the mean ± SEM. The Kruskal-Wallis with Dunn multiple comparisons test was used to determine the statistical significance of differences vs. wild-type animals fed live *E. coli* OP50, with brackets indicating the statistical differences between two specific conditions. * *p*<0.05**.** Right panel: Representative cropped images of *srh-234p::GFP* expression in the ADL cell body of adults. Images were acquired at the same exposure time. Bottom panel: Representative images of *acdh-1p::GFP* expression in adult animals at the indicated exposure times.

**Supplemental Figure 4: Vitamin B12 decrease *srh-234* expression but not in animals with mutations in the *pccb-1*, *pcca-1* and *hphd-1* genes.**

**(A-C)** Relative expression of *srh-234p::GFP* in the ADL cell body of adult animals with mutations in the canonical propionate breakdown pathway genes, *pcca-1* and *pccb-1* (propionyl-CoA carboxylase) **(A-B)** and the propionate shunt gene *hdpd-1* (3-hydroxypropionate-oxoacid transhydrogenase) **(C)** when fed either *E. coli* OP50 or *Comamonas aq.* DA1877 diets. Lower panels: Representative cropped images of *srh-234p::GFP* expression in the ADL cell body. **(D)** Percentage of adult animals showing wild-type dye-filling of ADL neurons when fed a *Comamonas aq.* DA1877 diet for the indicated mutant genotypes. **(E)** Relative expression of *srh-234p::GFP* in the ADL cell body of *nhr-68* mutants fed *E. coli* OP50 supplemented with or without 64nM Me-Cbl and/or 40mM propionate. **(A-D)** Representative images were acquired at the same exposure time for comparison. Data are represented as the mean ± SEM (n>20 animals). The Kruskal-Wallis with Dunn multiple-comparisons test was used to determine the statistical significance of differences vs. wild-type animals fed *E. coli* OP50, with brackets indicating statistical differences between two specific conditions and genotypes. ns, not significant, * *p*<0.05, ** *p*<0.01, *** *p*<0.001. Right panel: Representative cropped images of *srh-234p::GFP* expression in the ADL cell body. **(A-C, E)** Images were acquired at the same exposure time for comparison.

**
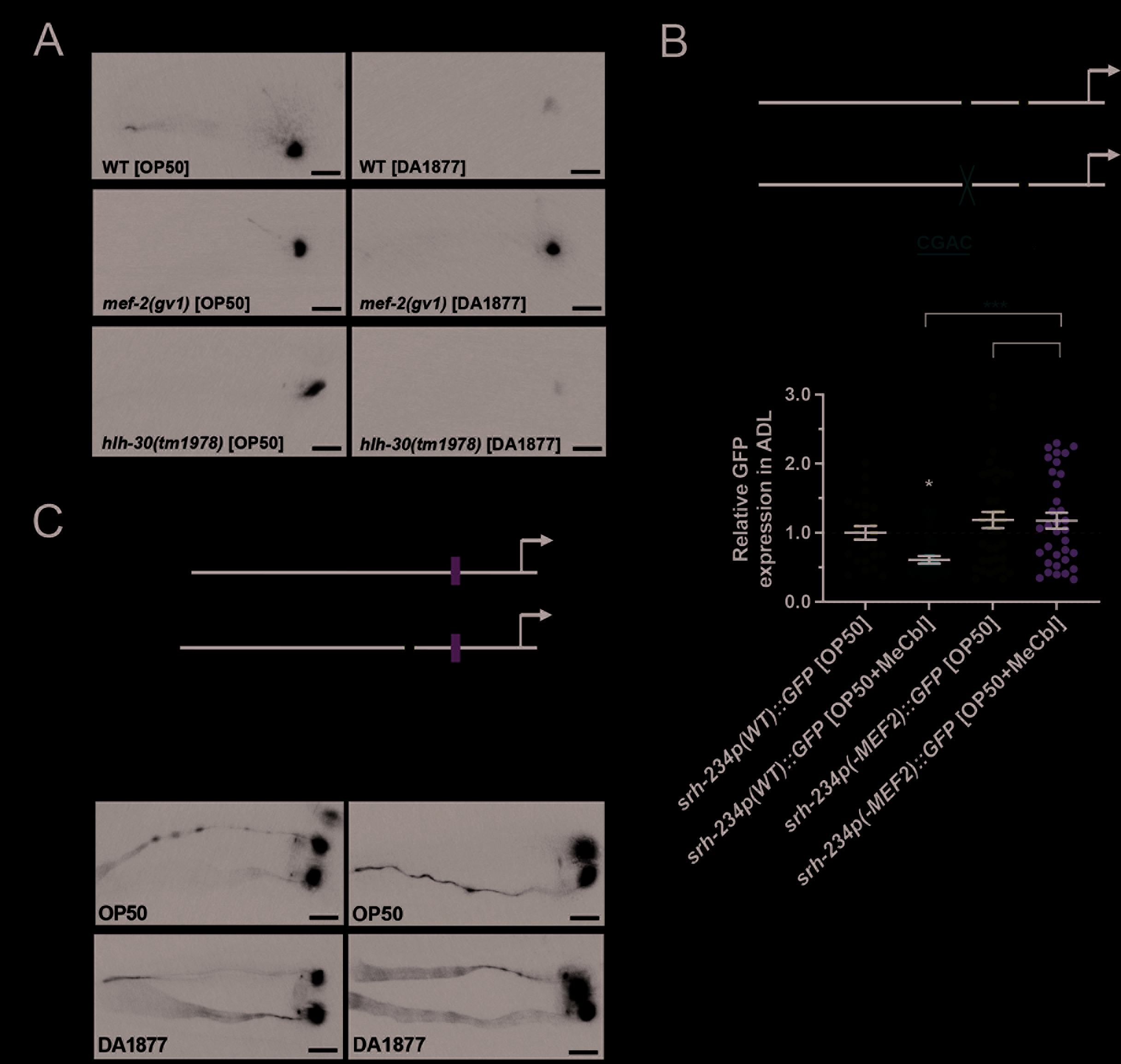
**

**Supplemental Figure 5: Introduction of the *srh-234* MEF2 site in the *cis*-regulatory region of *sre-1* does not confer regulation by vitamin B12.**

**(A)** Representative images of *srh-234p::GFP* expression in the ADL cell body of *mef-2* and *hlh-30* mutants fed on *Comamonas aq.* DA1877 compared to *E. coli* OP50 diets. Images were taken at the same exposure time (anterior is left). Scale is 15 µm. **(B)** Upper panel: The indicated lengths and positions of predicted regulatory elements relative to the translational start site of *srh-234* fused to the *gfp* coding sequence in an expression vector. Sequences in blue and purple indicate the predicted E-box motif and the mutated sequence in the core MEF2 binding site of *srh-234* (AGTTATATTTAA to AGTCGACTTTAA), respectively. Lower panel: Relative expression of *srh-234* in ADL driven by the wild-type *srh-234* promoter sequence (*srh-234p(WT)::GFP*) or with the mutated MEF2 binding site (*srh-234p(-MEF2)::GFP*) in adult animals fed *E. coli* OP50 supplemented with or without vitamin B12 (64 nM Me-Cbl final concentration). **(C)** Upper panel: The indicated lengths and positions of predicted regulatory elements relative to the translational start site of *sre-1* fused to the *gfp* coding sequence in an expression vector. Sequences in purple and green indicate the predicted E-box motif of *sre-1* with the artificially inserted MEF2 binding site sequence of *srh-234* near the *sre-1* E-box site, respectively. Lower panel: Representative expression of *sre-1* in ADL driven by the wild-type *sre-1* promoter sequence without (*sre-1p(WT)::GFP*) or with the *srh-234* MEF2 binding site inserted (*sre-1p(+MEF2)::GFP*) in adult animals fed on *Comamonas aq.* DA1877 compared to *E. coli* OP50. Images show both left and right cell bodies of ADL neurons (anterior is left), and were acquired at the same exposure time. Scale is 15 µm.
